# Identification of *cis*-Regulatory Elements in the Mammalian Genome: The cREMaG Database

**DOI:** 10.1371/journal.pone.0012465

**Published:** 2010-08-31

**Authors:** Marcin Piechota, Michal Korostynski, Ryszard Przewlocki

**Affiliations:** Department of Molecular Neuropharmacology, Institute of Pharmacology, Polish Academy of Sciences, Kraków, Poland; King's College London, United Kingdom

## Abstract

**Background:**

A growing number of gene expression-profiling datasets provides a reliable source of information about gene co-expression. *In silico* analyses of the properties shared among the promoters of co-expressed genes facilitates the identification of transcription factors (TFs) involved in the co-regulation of those genes. Our previous experience with microarray data led to the development of a database suitable for the examination of regulatory motifs in the promoters of co-expressed genes.

**Methodology:**

We introduce the cREMaG (*cis*-Regulatory Elements in the Mammalian Genome) system designed for *in silico* studies of the promoter properties of co-regulated mammalian genes. The cREMaG system offers an analysis of data obtained from human, mouse, rat, bovine and canine gene expression-profiling studies. More than eight analysis parameters can be utilized in user-defined combinations. The selection of alternative transcription start sites and information about CpG islands are also available.

**Conclusions:**

Using the cREMaG system, we successfully identified TFs mediating transcriptional responses in reference gene sets. The cREMaG system facilitates *in silico* studies of mammalian transcriptional gene regulation. The resource is freely available at http://www.cremag.org.

## Introduction

It is now estimated that more than 5% of the mammalian genome encodes functional information, including regions involved in the regulation of gene expression, whereas only 1.5% of the mammalian genome contains protein-coding information [1]. This estimation brings to light the importance of discovering information contained in the non-coding regions of the genome. Recently, there has been rapid growth in the amount of gene-expression-profiling data available, providing an almost unlimited wellspring of information about gene co-expression and co-regulation [2]. If the co-regulated genes share regulation pathways, their promoter regions are likely to share common properties [3]. Furthermore, the analysis of these common properties could allow for the identification of factors responsible for the regulation of the expression of particular sets of genes [4]. Such analyses include the identification of overrepresented transcription factor binding sites (TFBSs), regulatory modules or CpG islands. This approach provides novel insights into the molecular mechanisms controlling the process of gene transcription.

Methods for mining gene sequences for transcriptionally relevant information have become possible with the growing body of knowledge about mammalian genomes, gene expression and regulation of gene expression (See [5] for review). This growing body of knowledge has been transformed into multiple databases. The University of California, Santa Cruz genome browser (UCSC) and Ensembl databases contain whole-genome sequences and are sufficient for retrieving gene promoter sequences [6], [7]. However, more specific databases that are focused only on gene promoters, such as The Eukaryotic Promoter Database (EPD) or Cold Spring Harbor Laboratory mammalian promoter database (CSHLmpd), are also available [8], [9]. Retrieved promoter regions can be inspected for the presence and overrepresentation of TFBSs. The matrices for TFBSs can be found in the publicly available JASPAR database and in the partially publicly available TRANSFAC database [10], [11]. Furthermore, online tools, like CONREAL, are available for the discovery of TFBSs in conserved parts of gene promoters [12]. Finally, there are online tools based on the assumption that, if gene co-expression is controlled by one or more transcription factors (TFs), then the observed number of binding sites for those TFs should be greater than that expected by chance. Examples of such tools include oPOSSUM, PAP, TOUCAN2 and the Genomatix suite [4], [13], [14], [15].

However, there are some unresolved problems, and some areas await improvement. First, there are extreme differences in the information content among position weight matrices representing motifs of transcription factor binding sites, causing false-negative or false-positive matches [13]. Thus, the minimum relative score of matching position weight matrix used to report the position of a putative binding site (matrix score threshold) should not be identical for every matrix. Second, the conservation rate is not equal for every gene and its promoter [16]. Thus, the choice of criteria for determining conservation is one of the major problems of using phylogenetic footprinting [17]. Moreover, the phylogenetic footprinting conservation threshold should not be identical for every promoter. Also, it is well-established that there are genes with both constitutive and inducible transcriptional forms [18], [19]. However, there is an insufficient ability to choose among alternative promoters in current databases. Finally, tools for the inspection of quantitative promoter properties such as the GC-content or length of CpG islands are available [6], [20], [21]. However, insufficient data about CpG islands are integrated into tools that determine TFBS overrepresentation in sets of co-expressed genes.

Here, the new cREMaG (*cis*-Regulatory Elements in the Mammalian Genome) database is presented, which may help to resolve the above problems and allow for the discovery of TFs responsible for the regulation of co-expressed genes. Moreover, its successful application is demonstrated.

## Materials and Methods

### Annotation handling

The engine of the cREMaG database uses Ensembl Gene IDs as unique gene identifiers. For each of the *Mus musculus*, *Rattus norvegicus*, *Homo sapiens, Bos taurus* and *Canis familiaris* genes, we retrieved the Ensembl ID, Entrez ID, MGI or HGNC gene symbol, and the Affy ID from Ensembl using the BioMart interface [7], [22]. For each gene, a list of all known transcripts was obtained. All the transcripts for a particular gene were grouped into clusters of transcripts with the same transcription start site (TSS). Initial TSSs were retrieved from Ensembl. The Ensembl TSSs were remapped using Fantom 4 mappings of aggregations of cap-analysis gene expression (CAGE) tags [23]. First, the CAGE-tag mappings were remapped to the most recent genome assembly using the liftOver tool from UCSC [6]. Next, for each Ensembl TSS, we looked for the closest tag cluster in the range of 200 bp and took the CAGE tag starting site position within the transcriptional cluster supported by the highest number of CAGE tags as the representative TSS position. Finally, for every remapped TSS, we stored the maximum normalized tag count (tags per million, TPM) from all of the tissues analyzed by Fantom 4. If there was no CAGE tag for a particular Ensembl TSS, we stored -1 as the TPM value. The TPM values were further used as a measure of promoter strength.

### Sequence and conservation information retrieval

For every TSS, sequences 10 kbp upstream and 5 kbp downstream of the TSS (henceforth called the *promoter sequence*) and phastCons scores for multiple alignments of 30 vertebrate genomes were retrieved from UCSC.[6].

### Identification of CpG islands

The sequences 5 kbp upstream and 10 kbp downstream of every TSS were analyzed for the GC content and CG dinucleotide enrichment in frames of 200 bp. CpG islands were defined as sequences in which the length was >200 bp, the (G+C) content (%GC) was >50% and the ratio of observed to expected CpG dinucleotide frequencies (CpGobs/CpGexp) was >0.6 [24].

### Detection of TFBSs

Promoter sequences were scanned with TFBS matrices obtained from the JASPAR database and the public release of the TRANSFAC database using the TFBS BioPerl module [10], [25]. The matrix score, conservation score, distance from gene start and coding/non-coding values were assigned to every TFBS match. The data were stored in a relational database.

### Background precomputation

Three types of background sequences were prepared: random, core promoter and conserved promoter. To develop a random promoter background, 20 Mbp of random sequence was generated with equal numbers of all four nucleotides. To develop a core promoter background dataset, sequences containing the region 200 bp upstream of the gene start position for every gene in a genome were retrieved and concatenated. To develop a conserved promoter background, sequences with conservation scores higher than 75% for every gene were obtained and concatenated. The resulting random, core and conserved promoter sequences were scanned for all JASPAR and public TRANSFAC TFBSs at every integer matrix score threshold from 60% up to 100%. The frequencies at all thresholds were stored in a set of TFBS frequency tables.

### Optimization of matrix score threshold

Two user-defined parameters, *Random TFBS occurrence* and *Background sequence,* are combined with the TFBS frequency tables to define a matrix score threshold for each matrix. The threshold with the most similar (least-distant) background frequency (random occurrence) to the user-defined frequency in the user-defined type of sequence is set as optimal.

### Identification of overrepresented binding sites

TFBSs from all alternative promoters for a particular gene meeting the user-defined criteria are combined into one pool. This procedure is repeated for all genes from the query set. The total length of queried sequences filtered for user-defined parameters is computed. The fold-difference in TFBS frequency is computed by dividing the observed TFBS frequency by the background frequency. The probable number of genes with particular TFBS hit obtained by chance is computed. For each particular TFBS matrix, the fold-change in the frequency of genes containing a particular TFBS compared with a predicted background frequency is computed. Z-scores are computed based on the fold distribution for all TFBS matrices. The fold distribution is Gaussian. The p-value was defined as the probability of obtaining a specific range of z-scores using the standard normal distribution. The p-values for frequency fold and gene fold are computed separately. Moreover, a proportion p-value is computed, defined as the proportion of TFBS hits that can be explained by chance.

### Updating scheme

The cREMaG system was designed for easy and continuous updates. The Ensembl database, which is the sequence resource for the cREMaG database, is updated several times a year. A Perl script continuously retrieves and analyzes new sequences and orthologs and stores them in the MySQL database used by cREMaG. The *promoter properties* section of the database is updated daily. Adding a new batch of position weight matrices for TFBS requires computation of all genes in the database and thus requires about one month of computation. Therefore, the repository of TFBS matrices will be updated at least once a year.

## Results

### Usage and web interface

#### 1. Query step I – gene-set submission

**Figure 1:**
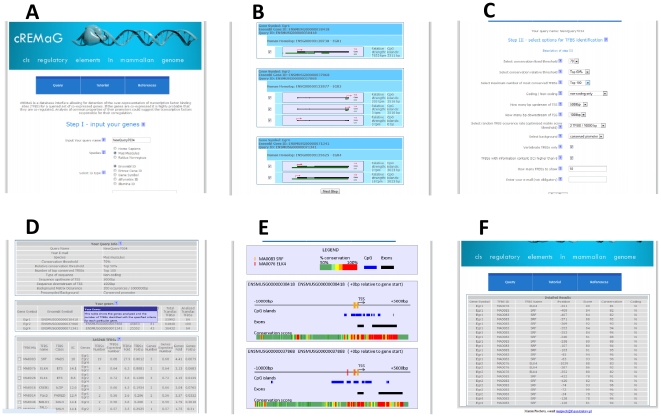
An overview of the graphical user interface. (A) The view of query step I – the gene-set submission in which the user is asked to input the query name and gene IDs and to select the species, type of Ids and the type of alternative TSSs. (B) The view of query step II – the alternative promoter selection in which the user is asked to confirm the identified genes and alternative promoters. (C) The view of query step III – the user is asked to select the analysis parameters. (D) Query step IV – the results overview. (E) cREMaG visualization of promoters. (F) Detailed table with particular transcription factor binding sites.

In step I, the user is asked to fill in the five-field form (Figure 1A). First, the query name may be passed. It is not obligatory, and if left blank, the system will generate a unique query name. Second, the species of the entered IDs should be selected. cREMaG currently supports five species: *Mus musculus*, *Rattus norvegicus*, *Homo sapiens, Bos taurus and Canis familiaris*. Five types of IDs are supported: Ensembl IDs, Entrez Gene IDs, gene symbols and Illumina and Affymetrix microarray IDs. It is recommended to use Ensembl Gene IDs because the engine of cREMaG is based on Ensembl IDs. Next, the IDs should be passed into the text field. It is recommended that users use between five and one hundred genes. Queries of fewer than five genes may yield unreliable results, and queries of more than 300 will slow down the analysis. Finally, the user may select whether to use all alternative TSSs, only the most distal TSSs, only the most highly expressed TSSs (based on TPM values) or TSSs with or without CpG islands.

The queried IDs are converted to Ensembl Gene IDs. Every identified Ensembl Gene ID is translated into a gene symbol. Next, cREMaG searches for all TSSs belonging to genes identified previously by the Ensembl Gene ID. If a gene symbol corresponding to more than one Ensembl Gene ID will be queried, all of the corresponding genes will be included in the analysis.

#### 2. Query step II – alternative promoter selection

In step II, the user is asked to confirm the identified genes and alternative promoters (Figure 1B). The results of the query IDs identification are presented in the form of small tables containing Ensembl Gene IDs with the gene symbol and query ID as headers and an internal table of all alternative TSSs containing promoter visualization, TPM values and lengths of CpG islands. The user can manually check or uncheck alternative TSSs using checkboxes.

#### 3. Query step III – analysis parameters setting

In step III, the user is asked to select the analysis parameters (Figure 1C). The *Conservation threshold* parameter is responsible for the elimination of TFBSs with conservation scores lower than the selected threshold. It is possible to select values from 60% (low conservation) to 90% (very high conservation). The *Top percent of conserved regions* parameter is responsible for the elimination of the TFBSs with the lowest conservation scores. It is possible to select values between 1% (only the most conserved TFBSs) and 100% (all TFBSs meeting other criteria). The *Maximum number of most conserved TFBSs* parameter was designed to analyze an equal number of TFBSs for every promoter independent of its conservation level. The *Coding/non-coding sequence* parameter allows users to search TFBSs in coding elements, non-coding elements or both. The *Length of upstream segment* parameter allows users to choose how many base pairs upstream of the TSS are used for the analysis (0 bp to 10,000 bp). The *Length of downstream segment* parameter allows the user to choose how many base pairs downstream of the TSS are taken for analysis (0 bp to 5000 bp). The *Random TFBS occurrence* (optimized matrix score threshold) parameter determines how restrictive the matrix score threshold should be set (starting from 1 TFBS/1,000,000 bp and ending at 1 TFBS/100 bp by chance). The *Random TFBS occurrence* parameter is combined with the *Select background* parameter to determine the background sequence type (random, core, promoter or conserved promoter), and together they determine the random TFBS occurrence rate.

#### 4. Query step IV – results overview

In the last step, the user is provided with five tables (Figure 1D). The first table shows which analysis parameters were selected. The second table shows the genes analyzed and the number of TFBSs identified with the specified criteria for each particular gene. The third table shows the fold differences of the most overrepresented binding sites from the JASPAR database. This table holds various types of information, including the TFBS matrix identifier, transcription factor name, transcription factor class and information content of matrix. The *Genes* column of the table contains the symbols of genes containing particular TFBS in their promoters. *TFBSs number* shows the total number of TFBSs found in all included promoters. *TFBSs Expected number* is the number of expected occurrences of a TFBS matrix and is computed based on the length of sequences used for the analysis and the random false-positive rate selected by the user. *TFBSs Fold* is the result of dividing the *TFBS number* by the *TFBSs Expected number*. The *TFBSs Fold p* is the p-value showing how the *TFBS fold* stands out from the fold distribution. *Genes Number* is the number of genes containing a particular TFBS. *Genes Expected Number* is the expected number of genes containing a particular TFBS. *Genes Fold* is the result of dividing the *Genes Number* by the *Genes Expected number*. The *Genes Fold p* is the p-value showing how *Genes Fold* stands out from the fold distribution. The *Proportion p-value* is the proportion of TFBS hits that can be explained by chance. The fourth table shows the fold-differences of the most overrepresented binding sites from the TRANSFAC database and contains the same information as the JASPAR table. The fifth table shows the average lengths of the CpG islands within the query set and within the whole genome and the p-value for this comparison.

### Examples of usage

#### 1. CREB-regulated gene-set

To demonstrate the functionalities of cREMaG, we utilized the results from gene expression profiling in the striatum of cocaine-treated mice lacking CREB in the forebrain, accompanied by the deletion of CREM[26]. We submitted Affy IDs of genes with striatal cocaine-dependent induction suppressed in *Creb1^Camkcre4^*; *Crem* ^–/–^ animals. The genes analyzed are widely recognized in the literature as immediate early genes (IEG) [27]. The cREMaG system was designed to analyze results coming from microarray data, and, therefore, annotations for the two frequently used microarray systems, Affymetrix and Illumina, were implemented. We submitted a list of 45 Affymetrix probe-sets for 30 CREB-regulated genes (see Table S1 in Supplementary Data) using default query options with a shorter promoter length (-1000 bp upstream, 0 bp downstream). An optimized matrix score threshold was developed to make it possible to compare different TFBS profiles at the same false-positive probability. CREB was correctly identified as a true-positive regulator of the queried gene-set (p<1*10^-4^, 1st rank). Moreover, SRF was also found (p = 0.010, 4th rank) as a true-positive regulator of IEG [28]. Another feature unique of the cREMaG database is the analysis of CpG islands. All genes from the CREB regulated gene set contain CpG islands within their promoters, with the average length of CpG islands much greater than the genomic average (p<1*10^-4^). The table with the query results is provided in the Table S2 in Supplementary Data.

#### 2. Alternative transcription start sites of the Homer1 gene

The cREMaG system was also designed to analyze the promoters of particular transcriptional variants. The *Homer1* gene has multiple transcript forms with distinct alternative TSSs. Homer1a is a CREB-inducible isoform of *Homer1* widely described in the literature, and its transcription start site is the most proximal from the beginning of the gene [29]. *Homer1* was submitted as a mouse gene symbol into the cREMaG system. In step II of the analysis, there was the possibility of selecting from four TSSs. The three cAMP response elements (CRE) were found only at the core promoter of the Homer1a isoform. This example shows the advantages of using the promoter of only the inducible transcriptional isoform when searching for overrepresented binding sites. While including all transcription start sites provides noise in the final results, the selection of particular promoters for the analysis may give a higher chance for true-positive results.

### Comparison with other tools

**Figure 2:**
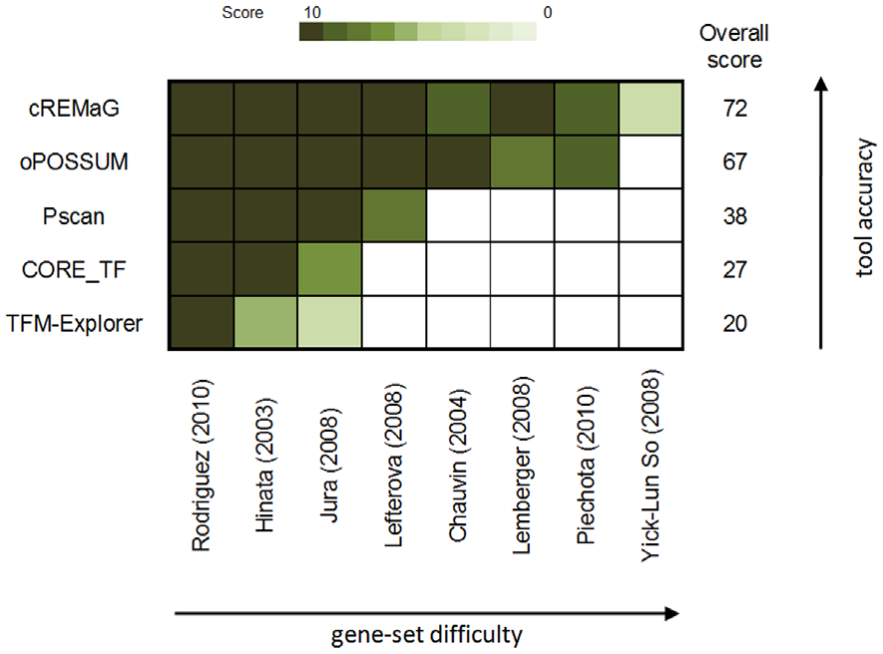
Comparison of cREMaG to other on-line tools. The heatmap plot presents the scores from cREMaG, oPOSSUM, CORE_TF, TFM-Explorer and Pscan for eight selected gene sets. The scores were calculated based on the rank in the results of the expected TFBS matrix: ten points for first rank (dark green color), nine points for second, down to one point for tenth rank (white color). The sum of the scores is presented on the right. The tools and gene sets are ordered by the sum of scores in decreasing order.

To demonstrate accuracy of cREMaG, we compared it with other available on-line tools: oPOSSUM, CORE_TF, TFM-Explorer and Pscan [4], [30], [31], [32]. For this purpose, we used eight reference gene sets: a) microarray profiling of cocaine-induced transcriptional alterations attenuated in SRF knockout animals with the expected motif being SRE [33]; b) microarray profiling of NF-κB-regulated genes in the human skin with the expected motif being Rel [34]; c) microarray profiling of cytokine-induced gene expression in human macrophages, with the expected motif being Rel [35]; d) ChIP-seq results including the 10 top-scoring PPARG binding sequences, with the expected motifs being PPARG or RXRA [36]; e) microarray profiling after pharmacological intervention for androgen-regulated genes in the epididymis, with the expected motif being AR [37]; f) microarray profiling of ethanol-induced genes inhibited by the co-administration of glucocorticoid receptor antagonist RU486, with the expected motif being NR3C1 [38]; g) microarray profiling of cocaine-induced transcriptional alterations attenuated in CREB knockout animals, with the expected motif being CRE [26]; and h) ChIP-chip results for glucocorticoid receptor-binding sequences, with the expected motif being NR3C1 [39]. Pscan and TFM-Explorer accept only RefSeq numbers. Thus, IDs (gene symbols, Affy IDs, and RefSeq IDs) from the selected gene sets were converted to RefSeq numbers using Ensembl BioMart. Sequences 1000 bp upstream of the most distal TSS were retrieved from Ensembl for use in CORE_TF. For the comparison, we used the default settings for all the tools including cREMaG. As a score, we used the rank of the expected motif in the obtained results, where ten points were given for the first rank, nine points for the second, down to one point for tenth rank. All of the tools got a score in three (a-c) out of the eight gene sets (Figure 2). cREMaG and oPOSSUM got a high score in the next four gene sets (d-g). In one gene set, only cREMaG got a score (h). The results obtained with cREMaG and oPOSSUM were similar. However, cREMaG received the highest summary score. All of the gene sets, with the original IDs, RefSeq numbers, promoter sequences and results, are provided in Table S3.

## Discussion

The cREMaG database was designed for the analysis of gene expression patterns obtained from microarray expression data. cREMaG allows for the identification of overrepresented TFBSs in the promoter regions of genes from co-expressed gene sets. Besides cREMaG, there are other systems suitable for the analysis of overrepresented transcription factor binding sites in mammalian genes, including oPOSSUM, PAP, CORE_TF, TFM-Explorer, Pscan and Genomatix [4], [13], [15], [30], [31], [32]. cREMaG requires only a web browser with no additional third-party plugins. No login is required. cREMaG can be tested with no presets or registration confirmation delay. Moreover, the resource is freely available for academic and commercial users.

Stable and well-established data repositories were selected to build cREMaG. cREMaG uses matrices from two publicly available TFBS matrices repositories, JASPAR and the public release of TRANSFAC [10], [11]. JASPAR has a relatively small set of matrices, but the redundancy of other larger sets of matrices like TRANSFAC or MatBase is very high [10], [15]. For example, there are six highly similar matrices for the CREB transcription factor binding site in TRANSFAC, but only one in JASPAR. This difference makes it difficult to compare the content of information in TFBS matrix repositories. However, TRANSFAC and MatBase contain some unique TFBS matrices, such as the matrix for PLZF in MatBase. However, cREMaG was designed to add new batches of TFBS matrices with ease and is not limited to any particular repository. As an alternative TSS repository, the Ensembl database was chosen [7]. Moreover, Ensembl TSSs were remapped using Fantom 4 CAGE tags [23]. cREMaG allows for the analysis of either selected alternative promoters or multiple TSSs at once.

Promoters stored in cREMaG were curated for their evolutionary conservation, based on the assumption that functional elements evolve more slowly than nonfunctional elements [12]. This assumption is only partially true [3]. However, including nonconserved promoter sections for analysis yields many more false positives for TFBSs, thus resulting in more noise in the analysis. The rates of evolution differ across genes and promoters [16]. Thus, the threshold for the conservation score should be selected in light of gene-specific characteristics. One of the attempts to resolve this problem is proposed in the oPOSSUM system [4], in which three conservation thresholds are linked with the appropriate maximum percentage of conserved regions, ranging from 10% to 30%. In cREMaG, the maximum percentage of conserved regions is unlinked to the other parameters and ranges from 1% to 100%. Moreover, highly conserved promoters have longer stretches of highly conserved regions, implicating a greater number of analyzed TFBSs and having a higher impact on the final result. Thus, the ability to choose the maximum number of conserved TFBSs was developed to allow all promoters to contribute equally to the final result. The maximal length of analyzed sequence varies widely across systems. In cREMaG, a maximal range of 15 kbp is possible for an analysis starting 10 kbp upstream of the TSS and finishing 5 kbp downstream of the TSS. This arbitrary range was selected as a compromise between possible noise coming from false positives on overly long sequences, a loss of true positives on overly short sequences and system efficiency. Moreover, if a gene contains multiple promoters, all are accessible for analysis at once. Specific sets of initiating dinucleotides are associated with different TSS types, and the surrounding GC content is well-correlated with the types of these dinucleotides [40]. Thus, cREMaG is also suitable for selecting only those TSSs that are surrounded by CpG islands.

Optimization of the matrix score threshold was resolved previously in the PATSER, MATCH and Genomatix tools [13], [41], [42]. Genomatix defines the optimized threshold of a weight matrix as the matrix similarity threshold that allows a maximum of three matches in 10 kbp of non-regulatory test sequences [13]. The MATCH software defines the optimized threshold of a weight matrix based on the number of matches in exonic sequences[42]. cREMaG defines the optimized threshold of a weight matrix as the threshold that allows a user-defined number of matches in 1 Mbp of user-defined background test sequences. For the identification of overrepresented binding sites in large gene sets, it is advisable to use more restrictive thresholds (a smaller number of random matches). If looking for possible gene targets of transcription factors, it is advisable to use less restrictive threshold, resulting in more false positives but fewer false negatives.

Our previous experience with microarray studies [35], [38], [43], [44] led us to develop the cREMaG system, which is suitable for the analysis of the regulatory properties of promoters of co-expressed genes. The system has novel features in addition to the well-constituted solutions that are implemented in some of the available free or commercial systems. The unique features of cREMaG include the optional selection of multiple or single alternative promoters for analysis linked with information about CpG islands, the maximum number of TFBSs per gene and the optimized level of restrictiveness of the matrix score thresholds linked with three distinct pre-compiled backgrounds. The cREMaG database constitutes a valuable resource for all researchers working with gene expression data. We aim to continuously import new data sources and update the database on a regular basis.

The database is freely available to academic and non-academic users at the http://www.cremag.org address. However, if you find the cREMaG database useful for your work, please cite this paper.

## Supporting Information

Table S1 List of Affy IDs of CREB-dependent genes from gene expression profiling in the striatum of cocaine-treated mice lacking CREB in the forebrain (Lemberger, 2008).
(0.02 MB XLS)

Table S2 Query results of CREB-regulated gene-set.
(0.02 MB XLS)

Table S3 The gene sets, with the original IDs, RefSeq numbers, promoter sequences and results of cREMaG comparison with other tools.
(0.60 MB XLS)
